# An Update on the Pathophysiology and Diagnosis of Inappropriate Secretion of Thyroid-Stimulating Hormone

**DOI:** 10.3390/ijms22126611

**Published:** 2021-06-21

**Authors:** Kenji Ohba

**Affiliations:** 1 Medical Education Center, Hamamatsu University School of Medicine, 1-20-1 Handayama, Higashi-ku, Hamamatsu, Shizuoka 431-3192, Japan; ohbak@hama-med.ac.jp; Tel./Fax: +81-53-435-2843; 2 Second Division, Department of Internal Medicine, Hamamatsu University School of Medicine, 1-20-1 Handayama, Higashi-ku, Hamamatsu, Shizuoka 431-3192, Japan

**Keywords:** thyroid function, inappropriate secretion of thyroid-stimulating hormone (IST), syndrome of inappropriate secretion of thyroid-stimulating hormone (SITSH), genuine IST

## Abstract

Inappropriate secretion of thyroid-stimulating hormone (IST), also known as central hyperthyroidism, is a clinical condition characterized by elevated free thyroxine and triiodothyronine concentrations concurrent with detectable thyroid-stimulating hormone (TSH) concentrations. Similarly, the term syndrome of IST (SITSH) is widely used in Japan to refer to a closely related condition; however, unlike that for IST, an elevated serum free triiodothyronine concentration is not a requisite criterion for SITSH diagnosis. IST or SITSH is an important indicator of resistance to thyroid hormone β (RTHβ) caused by germline mutations in genes encoding thyroid hormone receptor β (TRβ) and TSH-secreting pituitary adenoma. Recent evidence has accumulated for several conditions associated with IST, including RTH without mutations in the TRβ gene (non-TR-RTH), the phenomenon of hysteresis involving the hypothalamus-pituitary-thyroid axis (HPT-axis), methodological interference, and Cushing’s syndrome after surgical resection. However, little information is available on the systematic pathophysiological aspects of IST in previous review articles. This report presents an overview of the recent advances in our understanding of the etiological aspects of IST that are relevant for diagnosis and treatment. Moreover, the report focuses on the potential mechanism of IST caused by hysteresis in the HPT-axis (lagging TSH recovery) in terms of epigenetic regulation.

## 1. Introduction

Circulating concentrations of thyroid-stimulating hormone (TSH) and other thyroid hormones are tightly regulated in healthy individuals [1]. The liganded thyroid hormone receptor (TR) negatively regulates the synthesis and secretion of TSH in pituitary thyrotrophs, resulting in drastic decreases in serum TSH concentrations [2]. For example, the most common forms of hyperthyroidism, including Graves’ disease and toxic multinodular goiter, demonstrate elevated thyroid hormone levels with suppressed TSH concentrations.

**Figure 1:**
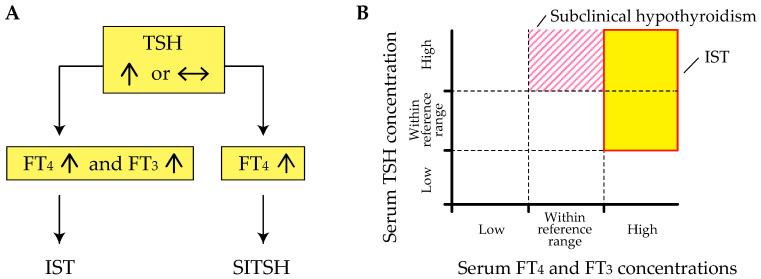
The different hormone profiles of inappropriate secretion of thyroid-stimulating hormone (IST) and syndrome of IST (SITSH). (**A**) A schematic explanation of the difference between IST and SITSH. Note that an elevated free triiodothyronine (FT_3_) concentration is a requisite diagnostic criterion for IST. (**B**) A schematic explanation of the difference between IST and subclinical hypothyroidism. The shaded region and striped region represent the areas of IST and subclinical hypothyroidism, respectively. FT_4_, free thyroxine.

Inappropriate secretion of TSH (IST) is characterized by the laboratory findings of elevated free thyroxine (T_4_) and triiodothyronine (T_3_) concentrations in the presence of detectable TSH concentrations [3,4], contrary to what happens in the most common forms of hyperthyroidism (Figure 1A). This abnormal pattern in the thyroid function test is an important hallmark of genetic and acquired disorders of the hypothalamus-pituitary-thyroid axis (HPT-axis), including resistance to thyroid hormone β (RTHβ) caused by germline mutations in genes encoding the β isoform of the TR (TRβ) and TSH-secreting pituitary adenoma (TSHoma). Although previous review articles have sufficiently investigated several conditions associated with IST, little information is available on systematic pathophysiological aspects of IST in previous review articles. In addition, a recent study has revealed that diagnostic delay and inappropriate treatments (e.g., pituitary surgeries in patients with RTHβ or thyroid ablation in patients with TSHoma) are still provided in several cases with thyroid hormone profiles suggestive of IST [5].

In this article, the author has performed a review of literature and has presented a balanced overview of the recent advances in our understanding of the clinical, biochemical, and etiological aspects of IST relevant for its diagnosis.

## 2. Definition of IST

The term “IST” was proposed by Gershengorn and Weintraub in 1975 [6]. In a subsequent report, IST was described as a condition characterized by elevated serum levels of immunoreactive TSH in the presence of elevated free thyroid hormone concentrations [3]. However, it has been suggested that TSH secretion is compensatory and appropriate in RTHβ, because the response to thyroid hormones in peripheral tissues is reduced due to impairment of the TR [7]. Beck-Peccoz et al. also suggested that the definition of “IST” appears inadequate, as it does not reflect the pathophysiological events underlying TSHoma and RTHβ [4]. Therefore, the authors proposed the term “central hyperthyroidism” instead of IST, because TSH itself is responsible for the hyperstimulation of the thyroid gland and the consequent thyrotoxicosis.

The term “syndrome of inappropriate secretion of thyroid-stimulating hormone (SITSH)” has been widely used in journal articles and textbooks written by Japanese researchers since the late 1980s. Based on the 2016 Japan Thyroid Association (JTA) guidelines for the diagnosis of RTHβ, SITSH is defined as being characterized by elevated free T_4_ and normal-to-elevated TSH concentrations [8] (Figure 1A). An elevated serum free T_3_ concentration is not a requisite criterion for SITSH diagnosis. In this study, the author conducted a MEDLINE search using the keyword “SITSH” to retrieve relevant articles published between 1975 and 2020. The literature search yielded 30 articles, of which 28 were published in Japanese institutions.

Physicians must keep in mind that elevated thyroid hormone concentrations are requisite for the diagnosis of IST, because at least two previous studies defined the laboratory findings of inappropriately elevated TSH concentration concurrent with non-elevated thyroid hormone concentrations as the criterion for IST [9,10]. Tyrosine kinase inhibitor therapy, specifically therapy using axitinib, can reportedly cause inappropriately elevated TSH concentrations despite the thyroid hormone concentrations being within the reference range [11]. Similar patterns of thyroid function profiles have been reported in patients with macro-thyrotropin (macro-TSH) [12,13,14], or in those with RTHβ and Hashimoto’s thyroiditis [15]. These findings of thyroid function tests meet the definition of subclinical hypothyroidism but not IST (Figure 1B).

## 3. Pathophysiology of IST

### 3.1. Overview

Sufficient evidence has been accumulated for several conditions associated with IST, as summarized in Table 1. In a recent study reported by Campi and colleagues [5], the term “genuine IST” was used to indicate the etiology of IST, including TSHoma, RTHβ, and a syndrome clinically and biochemically indistinguishable from RTHβ but without mutations in the TRβ gene (non-TR-RTH). Accordingly, pituitary TSHoma, ectopic TSHoma, RTHβ, and non-TR-RTH were categorized as the causes of genuine IST in this review article.

### 3.2. Causes Associated with the Classification Proposed by Gershengorn and Weintraub

Gershengorn and Weintraub proposed a classification of IST in terms of its pathogenesis [6]. The major classification was dependent on whether IST was either associated with a neoplasm (class I) or was not (class II). Neoplastic IST (class I) was further classified based on tumor location (i.e., class I-A; pituitary tumors and class I-B; non-pituitary tumors (ectopic production)). Non-neoplastic ISTs (class II) were classified into three groups: RTH (class II-A), abnormal stimulation of TSH secretion by thyrotropin-releasing hormone (TRH) or other stimulators (class II-B), and defective suppression of TSH secretion by somatostatin, dopamine, or other suppressors (class II-C).

When Gershengorn and Weintraub proposed their classifications, clinical entities of I-A and II-A had been documented, but other classes were only postulated [6]. Subsequent studies have revealed that several conditions or situations are associated with IST, as proposed by the authors.

First, the initial case of IST caused by ectopic TSHoma was reported in 1996 [16]. To date, more than ten similar cases have been documented [17].

Second, Refetoff et al. proposed the term impaired sensitivity to thyroid hormone (ISTH), which includes RTHβ, non-TR-RTH, selenocysteine insertion sequence-binding protein 2 (SBP2) defect, monocarboxylate transporter 8 (MCT8) defect, and RTHα caused by germline mutations in the genes encoding the α isoform of the TR (TRα) [18]. Among them, the hormonal profile of non-TR-RTH fulfills the definition of IST. In contrast, thyroid function profiles of SBP2 defects, MCT8 defects, and RTHα do not meet the definition of IST, because they are associated with high serum T_4_ or T_3_ only concurrent with non-suppressed TSH concentrations [7,19].

Third, Kaplan et al. previously reported that continuous TRH treatment resulted in modest sustained increases in both serum TSH and thyroid hormone concentrations in patients with amyotrophic lateral sclerosis [20]. The authors suggested that the hypersecretion of TRH from the hypothalamus is responsible for some cases of IST. In this context, we previously described the case of a patient with SITSH coexisting with thymoma-related peripheral nerve hyperexcitability, in which excessive secretion of TRH from the paraventricular nucleus seemed to be induced by antibodies against voltage-gated potassium channels [21]. These conditions can be classified as class II-B. In contrast, recent pharmacological studies have reported several TRH analogs possessing potent neuropharmacological activities despite weak pituitary stimulation [22]. For example, a TRH analog, taltireline hydrate, was reported to induce no significant effects on the HPT-axis in patients with brain stroke [23].

Fourth, Tamada et al. have recently reported that patients with Cushing’s syndrome frequently develop SITSH after surgery [24]. The authors suggested that the rapid decrease in glucocorticoid concentration after surgical resection was responsible for the development of SITSH. This disease can be classified as a class II-C condition.

### 3.3. IST Associated with Methodological Interference and Phenomenon of Hysteresis

In addition to the pathophysiology of IST as proposed by Gershengorn and Weintraub [6], other conditions have been reported to cause laboratory findings resembling IST, including methodological interference and the phenomenon of hysteresis in the HPT-axis (lagging TSH recovery).

First, several studies have revealed the detailed mechanisms of methodological interference, such as heterophilic interference, thyroid hormone autoantibodies, or assay-specific interference [25,26]. It has also been reported that familial dysalbuminemic hyperthyroxinemia (FDH), an autosomal dominant disorder, resembles SITSH via methodological interference [27,28]. Because patients with FDH have abnormal human serum albumin (HSA) with an increased binding affinity to T_4_, a relatively larger quality of abnormal HSA-bound T_4_ appears to be measured as free T_4_ during the analytic processes [28]. Detailed information on the mechanism of methodological interference has been discussed elsewhere [25].

Second, in the phenomenon of hysteresis in the HPT-axis (lagging TSH recovery), persistent TSH elevation (or suppression) with consequent lagging of thyrotroph recovery following hypothyroidism (or thyrotoxicosis) has been documented for many years [29]. Delayed response of thyrotrophs could result in non-suppression of TSH despite elevated thyroid hormones in several conditions (e.g., pre-existing hypothyroidism secondary to chronic thyroiditis and subsequent onset of destructive thyroiditis, or levothyroxine replacement therapy associated with poor compliance). Here, we focus on the potential mechanism of this condition in terms of epigenetic regulation.

### 3.4. Possible Mechanism of Hysteresis Involving the HPT-Axis

**Figure 2:**
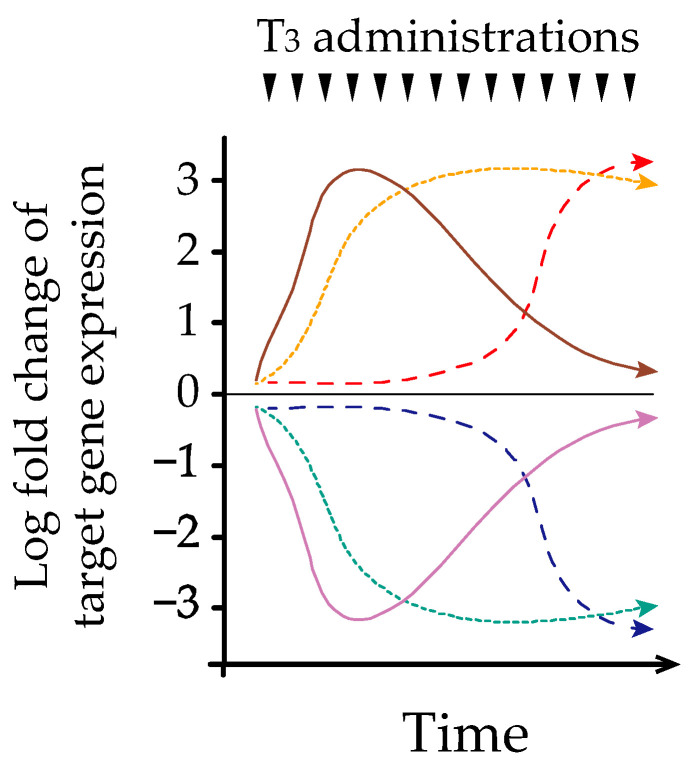
Schematic explanation of three differential expression patterns of both positively and negatively regulated thyroid hormone-responsive genes during chronic triiodothyronine (T_3_) treatment. Gene expression microarrays were performed on liver harvested from mice before and during chronic T_3_ treatment [33]. Both positively and negatively regulated T_3_ responsive genes were identified that could be divided according to their characteristic into three general types: genes regulated only acutely by T_3_ (solid line), those regulated only chronically by T_3_ (dashed line), and those regulated by both treatments (dotted line). Arrowheads represent liothyronine injections.

Hysteresis refers to the phenomenon observed in systems exhibiting a memory effect, such that the response to an input is delayed by a lag time [30]. Melvin et al. first described hysteresis involving the HPT-axis in 2007 [31]. However, the precise mechanism underlying this phenomenon remains poorly understood. We and others have identified early, late, and sustained patterns of hepatic transcriptional responses after acute and/or chronic thyroid hormone treatment in adult murine models [32,33] (Figure 2). Further analysis of epigenetic regulation demonstrated that histone H3 acetylation at lysines 9 and 14 (H3K9/K14ac) was associated with acute thyroid hormone stimulation, whereas histone H3 trimethylation at lysine 4 (H3K4me3) is associated with chronic stimulation [33]. Similarly, Umezawa et al. reported that chronic thyroid hormone treatment in a rat pituitary cell line caused sustained suppression of TRH, which was associated with a prolonged decrease in H3K4me3 and only a transient reduction in H3K9/K14ac [34]. Based on these findings, different chromatin modifications of target genes could play an important role in regulating transcription during acute and chronic thyroid hormone treatments. We have also reported that thyroid hormones reduce hepatic TRβ protein expression in a time-dependent manner [35]. Taken together, time-dependent changes in transcriptional mechanisms may partly explain the hysteresis of thyroid hormone-responsive genes. Further studies are needed to clarify the mechanism of hysteresis associated with the HPT-axis, especially using the pituitary and hypothalamic models.

## 4. Epidemiology of IST

The precise prevalence of IST remains unknown. We have retrospectively analyzed 8,183 concurrent measurements of TSH and free T_4_ in individuals previously examined using a one-step platform in a community hospital [14]. The results showed that the thyroid hormone profiles of 178 (2.2%) individuals fulfilled the diagnostic criteria for SITSH. Importantly, the frequency of genuine SITSH has been reported to be relatively low, ranging from 15% to 20% in all cases that fulfilled the diagnostic criteria of SITSH [36,37]. Regarding the percentage of each cause of genuine IST, Maccia et al. examined a large patient cohort of 99 consecutive cases, wherein RTHβ, non-TR-RTH, and TSHoma were determined to be 52, 16, and 31, respectively [38].

Several studies have described the epidemiology of causative disorders of IST. First, the incidence of RTHβ was estimated to be 1 case per 40,000 live births, according to limited results from T_4_-based neonatal screening programs [39,40]. Because RTHβ is rarely identified by TSH-based routine neonatal screening programs, the precise incidence of RTHβ is unknown. Second, TSHomas account for 0.5–3% of all pituitary adenomas, and the prevalence in the general population is around one case per million [41]. Interestingly, Teng identified an extremely rare case of TSHoma coexisting with RTHβ [42]. Third, the prevalence of FDH varies depending on ethnic origin, with the highest occurring in Hispanics [43]. Decosimo screened approximately 280 families and detected FDH in four families (incidence, 1.4%) [44]. In contrast, the prevalence of FDH is considered to be 1 in 7000 to 1 in 16,000 neonates in the Caucasian population [45]. The frequency was reported to be much lower in Japan, with 1 case per 83,000 live births [40].

## 5. Clinical Characteristics of IST

The clinical presentation of IST depends on the underlying disorders. First, most patients with TSHoma present with signs and symptoms of hyperthyroidism that are frequently associated with pressure effects of pituitary adenomas [46]. A single-center study of 90 consecutive cases of TSHoma in Japan identified goiter and palpitation (or tachycardia) as the two most common signs or symptoms [47]. Second, the signs and symptoms of RTHβ or non-TR-RTH are heterogeneous because the degree of compensation to tissue hyposensitivity due to elevated thyroid hormone concentrations varies among individuals as well as in different tissues. The two most common clinical features of RTHβ include goiter and palpitations [7,46]. Third, individuals with IST caused by methodological interference were asymptomatic.

## 6. Differential Diagnosis of IST

### 6.1. Confirmation of Genuine IST

The initial step of differential diagnosis is to confirm genuine IST. First, attention should be paid to IST caused by assay interference whenever clinical or biological discrepancies arise. Medical interviews and physical examinations are important because individuals with these conditions are asymptomatic. Repeated thyroid function tests with different assay systems (e.g., the one-step or two-step platforms) have been recommended for suspected cases [5,8,41,48]. In addition to the comparison of assay methods, dilution procedures, polyethylene glycol (PEG) precipitation test, and gel filtration chromatography would be helpful in excluding IST caused by assay interference [25]. Because these methods are not routinely performed outside the academic setting, the physician may communicate with the manufacturers and/or laboratory departments of each institute regarding this. Second, IST due to the hysteresis of the HPT-axis should be ruled out by clinical history taking and/or repeated thyroid function tests at different time points. For example, the 2016 JTA Guideline recommends measurements at one and three months after the initial assessment [8]. A case of RTHβ presenting occasional thyroid hormone profiles of SITSH was recently reported [49]. The authors speculated that some small occasional variations in thyroid function profiles, such as those usually experienced in the healthy population, occurred near the borderline between the area within reference ranges and that of SITSH. More recently, Okuma reported the first case of TSHoma, wherein the thyroid hormone profile occasionally demonstrated SITSH [50]. Accordingly, physicians must keep in mind that not only IST associated with the hysteresis of the HPT-axis, but also etiologies of genuine IST, could demonstrate occasional thyroid hormone profiles of IST. Lastly, IST caused by TRH administration and Cushing’s syndrome after surgical resection could be excluded by medical interview.

### 6.2. Differential Diagnosis of Genuine IST

Once the existence of genuine IST is confirmed, it is important to differentiate between RTHβ, non-TR-RTH, and TSHoma [5]. The possible presence of neurological signs and symptoms associated with the pressure effects of the pituitary adenoma or clinical features of concomitant hypersecretion of other pituitary hormones supports the probability of pituitary TSHoma occurrence. Because no differences in the basal concentrations of TSH and free thyroid hormones have been reported [38,41], several diagnostic steps are required. First, investigating the presence of IST in the family members of a patient can provide important clues for the diagnosis of RTHβ, because no familial cases of TSHoma have been documented [46]. Notably, the frequency of de novo RTHβ cases is approximately 20% [7]. Second, genetic testing and imaging studies are useful to confirm the diagnosis of RTHβ and TSHoma, respectively. However, differential diagnosis may be challenging if the pituitary adenoma is very small or missing in patients with the absence of mutations in the TRβ gene. Patients with IST and negative pituitary findings may develop TSHoma during long-term follow-up, as Campi et al. recently reported that pituitary MRI returned negative results in 6 of 26 cases with TSHoma [5]. The differential diagnosis between non-TR-RTH and TSHoma is important in some patients with pituitary adenoma, because approximately 10% of the healthy adult population has pituitary abnormalities on MRI scans that are compatible with the diagnosis of asymptomatic pituitary adenomas [51]. A recent study revealed that 11 out of 45 patients with RTHβ have small pituitary lesions [5].

### 6.3. Differential Diagnosis in Problematic Cases of IST

Several procedures for the differential diagnosis of problematic cases of IST have been proposed, including dynamic testing and determination of biochemical parameters. In line with dynamic testing, it has been suggested that the combination of dynamic testing could increase the accuracy of the diagnostic workup [41,48]. First, the TRH stimulation test demonstrated normal or increased TSH responses (similar to hypothyroid) in most patients with RTHβ or non-TR-RTH, whereas the vast majority of patients with TSHoma do not respond to TRH [38,46,47]. Second, the T_3_-suppression test (fixed dose of 75–100 μg of liothyronine (L-T_3_) administered for 7–10 days [37,46]) is useful to evaluate the presence of TSHoma, because TSH concentrations after T_3_-suppression are reportedly higher in cases with TSHoma than in cases with RTHβ [5,52]. Third, a standardized diagnostic protocol using incremental doses of L-T_3_ [53], instead of a fixed dose is important for the diagnosis of RTHβ and non-TR-RTH, wherein blunted TSH suppression after TRH administration and diminished metabolic response are typically observed [7]. However, dynamic tests using fixed or incremental doses of L-T_3_ are time-consuming and contraindicated in elderly patients or in those with coronary heart disease [52]. Fourth, attention has been paid to dynamic tests using somatostatin analogs (SSAs). Mannavola identified a significant reduction in serum thyroid hormone concentrations after the long-term administration of SSA in patients with TSHoma but not in those with RTHβ [54]. More recently, Han et al. demonstrated that even a short-term dynamic test using short-acting SSAs may provide an alternative diagnostic approach for TSHoma [55].

The measurement of biochemical parameters is also important to reach a differential diagnosis in problematic cases of IST. Previous studies have revealed that serum glycoprotein hormone α-subunit (α-GSU) concentrations are elevated in more than 70% of patients with TSHoma, particularly in those with a macroadenoma [56], whereas this parameter is within the reference range in most patients with RTHβ or non-TR-RTH [38,46]. As with other biochemical parameters, measurement of metabolic markers of thyroid hormone action can be helpful in determining peripheral resistance to thyroid hormones. Among these markers, serum sex hormone-binding globulin (SHBG) and carboxy-terminal cross-linked telopeptide of type-I collagen (ITCP) appear to be useful for differential diagnoses [41,48]. Other circulating markers, including alkaline phosphatase, brain natriuretic peptide, cholesterol, creatine kinase, and ferritin are of limited help in assessing the impact of thyroid hormones on peripheral tissues [38,41,57].

For a better understanding of the differential diagnosis of problematic cases of IST (or SITSH), several flowcharts have been proposed [5,8,41,48].

## 7. Treatment

Treatment for genuine IST depends on the causative disorders. Recent reviews have described details of the treatment for RTHβ and TSHoma [7,46].

The initial treatment for TSHoma requires preoperative medical therapy to control both thyroid function and pituitary tumor size [41]. Once euthyroidism is achieved, transsphenoidal pituitary adenomectomy is recommended. Pharmacotherapy was selected for patients who could not undergo surgery or who are not able to achieve remission after surgery, wherein the administration of long-acting SSA (e.g., lanreotide or octreotide) is generally recommended [58].

Treatment is not needed in most patients with resistance to RTHβ because its hyposensitivity appears to be adequately compensated by the increased free T_4_ and free T_3_ concentrations [59]. Unnecessary treatments, such as antithyroid drugs, thyroidectomy, or radioactive iodine, would lead to worsening of symptoms in patients with compensated RTHβ. However, when RTHβ is not compensated, levothyroxine can be administered in incremental doses with simultaneous monitoring of thyroid hormone metabolic markers. Because impaired responsiveness to thyroid hormone was mediated by mutated TRβ but not TRα, TRβ-specific analogs that have a higher affinity for TRβ than for TRα may be a candidate choice in the future. Additionally, because TRα, rather than TRβ, is dominantly expressed in the heart, some patients develop symptoms of thyrotoxicosis, especially tachycardia and tremor, and are usually responsive to the administration of β-adrenergic blockers. Non-TR-RTH management was identical to that of RTHβ.

Primary thyroid disorders or assay interference is relatively frequent and may cause additional uncertainty in management of IST. For example, concurrent primary hypothyroidism, primary thyrotoxicosis, and macro-TSH have been reported in patients with RTHβ [15,60,61,62]. In this regard, Campi et al. demonstrated that concurrent autoimmune thyroiditis was diagnosed in 19.2% (5/26) of patients with TSHoma and 34.4% (21/61) of patients with RTHβ [5]. Little is known about the management of patients with RTHβ or TSHoma who develop a second thyroid disorder. The diagnosis and treatment of these conditions are important issues that need to be addressed further.

## 8. Conclusions

In conclusion, IST is a clinical condition characterized by the hormonal profile of elevated free T_4_ and T_3_ concentrations in association with the presence of non-suppressed TSH concentrations. This condition is an important indicator of genetic and acquired disorders of the HPT-axis, including RTHβ and TSHoma. Early diagnosis of IST is important to prevent inappropriate management. Further studies to evaluate the optimal diagnostic procedures may improve the outcomes of patients with IST.

## Figures and Tables

**Table 1:** Possible causes of inappropriate secretion of thyroid-stimulating hormone (IST).

1. Causes associated with the classification proposed by Gershengorn and Weintraub [6] I. Neoplastic production of thyroid-stimulating hormone (TSH) Thyrotropin-secreting pituitary adenoma (TSHoma) * Ectopic TSHoma * II. Non-neoplastic pituitary hypersecretion of TSH Resistance to thyroid hormone β (RTHβ) * Non-TR-RTH * Thyrotropin-releasing hormone (TRH) administration Cushing’s syndrome after surgical resection 2. Methodological interference Heterophilic interference Thyroid hormone autoantibodies Assay-specific interference Familial dysalbuminemic hyperthyroxinemia (FDH) 3. Hysteresis involving the hypothalamus-pituitary-thyroid axis (lagging TSH recovery)

* indicates causes of genuine IST based on the findings reported by Campi and colleagues [5]. Non-TR-RTH, RTH without mutations in the thyroid hormone receptor β gene.

## Data Availability

The data that support the findings of this study are available from the corresponding author upon reasonable request.

## Abbreviations

FDH	Familial dysalbuminemic hyperthyroxinemia
HPT-axis	Hypothalamus-pituitary-thyroid axis
HSA	Human serum albumin
IST	Inappropriate secretion of thyroid-stimulating hormone
JTA	Japan Thyroid Association
L-T_3_	Liothyronine
Macro-TSH	Macro-thyrotropin
MCT8	Monocarboxylate transporter 8
Non-TR-RTH	Resistance to thyroid hormone without mutations in the thyroid hormone receptor β gene
RTHβ	Resistance to thyroid hormone β
SBP2	Selenocysteine insertion sequence-binding protein 2
SITSH	Syndrome of inappropriate secretion of thyroid-stimulating hormone
SSA	Somatostatin analog
T_3_	Triiodothyronine
T_4_	Thyroxine
TR	Thyroid hormone receptor
TRα	α isoform of thyroid hormone receptor
TRβ	β isoform of thyroid hormone receptor
TRH	Thyrotropin-releasing hormone
TSH	Thyroid-stimulating hormone
TSHoma	Thyrotropin-secreting pituitary adenoma
